# Barriers and Enablers to Young People’s Posting, Responding, and Reading Behaviors on Mental Health Forums Using the Behavior Change Wheel: Qualitative Study

**DOI:** 10.2196/71549

**Published:** 2025-10-31

**Authors:** Zhen Lim, Si Yin Lim, Siqi Lu, Leslie Morrison Gutman

**Affiliations:** 1 University College London London United Kingdom

**Keywords:** internet, online, adolescents, discussion board, evaluation, qualitative, survey

## Abstract

**Background:**

Web-based mental health forums have the potential to play a significant role in providing accessible support for young people, supplementing in-person services and contributing positively to their mental well-being. However, limited engagement often constrains their impact and effectiveness in supporting young people.

**Objective:**

Using the Behavior Change Wheel framework, this qualitative study explores the barriers and facilitators to young people’s engagement with web-based mental health forums, focusing on the behaviors of creating new posts, responding to posts, and reading posts. Behavior change techniques (BCTs) are identified to address these barriers.

**Methods:**

Semistructured interviews were conducted with 13 young people aged 17-25 years who use UK-based youth mental health forums. Three participants self-identified as men, 8 as women, 1 as nonbinary, and 1 chose not to disclose their gender. Transcripts were coded using the Theoretical Domains Framework (TDF), followed by inductive theme generation. TDF barriers were then mapped to BCTs to suggest intervention strategies.

**Results:**

Thematic analysis revealed ten inductive themes across 5 TDF domains. Of these, 3 were enablers, 2 were barriers, and 5 functioned as both enablers and barriers. The findings indicated that skills, beliefs about consequences, emotions, and the social and physical environment are key influences on young people’s engagement with web-based mental health forums. Positive emotions experienced after using the forums enabled posting, responding, and reading behaviors. Enablers of more active participation (ie, posting and responding) included anonymity and positive interactions with other users. The presence of moderators acted as an enabler for all 3 behaviors by providing a safe environment, but also as a barrier to posting, as moderation could restrict the content of users’ posts. Similarly, mobile access facilitated posting, responding, and reading, whereas layouts not optimized for mobile use acted as barriers to typing and reading on the go.

**Conclusions:**

This study contributes to the existing knowledge base by examining the different ways in which young people engage with youth mental health forums. Different strategies may be prioritized and adopted depending on whether forum providers aim to increase more active forms of engagement (eg, posting and responding, which can be encouraged by fostering positive interactions with other users) or overall engagement (eg, establishing clear rules of engagement and optimizing web page content for mobile access can benefit all forms of engagement). These insights can help improve the delivery of youth mental health forums and foster a positive ecosystem of support for young people.

## Introduction

### Young People’s Mental Health and the Role of Web-Based Forums

Globally, young people are experiencing a significant decline in mental health, with rising levels of reported mental health problems [1,2]. The COVID-19 pandemic, in particular, accelerated the deterioration of young people’s mental health [3]. In the United Kingdom, 1 in 3 young people aged 18-24 report symptoms of anxiety, depression, or both—an increase since 2000, when fewer than 1 in 4 (24%) reported these problems [4]. However, a recent report identified significant gaps in early mental health support for young people, with a rising number reaching crisis point [5]. From 2022 to 2023, the Children’s Commissioner’s annual report on children’s mental health [6] found that 28% of children and young people referred to mental health services were still waiting for support, with some waiting up to 2 years.

Viable stopgaps for young people include web-based channels such as social media or mental health forums, which are typically available 24/7 and can potentially provide accessible mental health support at scale. As digital natives, young people are adept at using the internet to seek support and are often favorable toward this approach, as it offers greater convenience and anonymity [7]. While social media may have a wider reach [8], mental health forums can be seen as a more focused and structured channel for young people to obtain mental health support [9]. Engaging in youth mental health forums can offer benefits such as emotional support, alleviation of feelings of isolation and stress, and reductions in symptoms of depression and self-harm [10,11]. However, potential drawbacks include increases in mental distress and self-harm tendencies, particularly when safeguards are insufficient in forums addressing sensitive or distressing topics [9]. Thus, it is important to continually explore ways to increase the effectiveness of these forums while reducing their potential harmful impacts. In addition, young people’s low engagement in web-based mental health forums remains a challenge, potentially limiting their effectiveness [12]. Research shows that mental health forums often fail to achieve their intended purpose because of low engagement rates [13], and users are unlikely to continue participating in inactive forums due to the limited support available [14]. Sustaining user activity in web-based forums helps to build a pool of knowledge and ensure that the forum remains relevant, facilitates the creation of online connections, and provides necessary support [14,15]. Therefore, there is a need to better understand the factors influencing engagement to maximize potential benefits.

Research shows that young people engage in web-based mental health forums in various ways. Many studies dichotomize them into *posters*, who are active participants contributing most of the content, and *lurkers*, who are passive participants and read posts without contributing [16,17]. Posters may create initial posts that introduce topics for discussion, often focusing on their own concerns, questions, thoughts, and lived experiences [18]. They may also respond to others’ posts, providing emotional or informational support to the user who shared the original comment [19]. Lurkers, on the other hand, read posts either to find specific information or to browse more generally [20]. Young people’s engagement in web-based mental health forums therefore encompasses multiple behaviors, including posting original content, responding to others, and reading posts.

While these behaviors should be treated as distinct, as they have different motivating and hindering factors [21], existing studies often examine youth mental health forum use more generally [22,23] or focus only on posters and lurkers [15,16]. However, this limits our understanding of the factors influencing young people’s engagement across a range of different behaviors. Using the Behavior Change Wheel (BCW) approach [21], this qualitative study explores the barriers and enablers to young people’s engagement in mental health forums, focusing on posting, responding, and reading behaviors, and identifies strategies for improvement. Such an examination can reveal both unique and shared influences and support the development of targeted interventions, offering a holistic view of young people’s engagement with youth mental health forums.

### Research Background

Current research has highlighted the benefits of mental health forums for young people. Web-based forums foster a sense of community and friendship, providing spaces where young people can receive practical advice on a variety of topics [18]. They not only offer informal support but also signpost young people to formal support services when necessary [24,25]. Thus, they can serve as important conduits, facilitating access to relevant professional services and encouraging help-seeking behavior among young people who need it. Furthermore, unlike professional or in-person services, web-based forums connect young people around the clock and therefore serve as valuable supplements to other mental health services within the broader ecosystem [19].

The characteristics of web-based mental health forums also enable different types of engagement among young people. As these forums contain a wealth of information and messages that are typically archived for long periods, young people can access relevant content at any time [9]. Reading the repository of existing discussion threads therefore provides immediate, personally relevant, and actionable information [14], such as personal experiences on managing specific situations and techniques for improving one’s own mental health [26,27]. Reading posts also allows young people to reflect on their personal difficulties through the lens of others’ experiences, for instance, by reframing common challenges as manageable [28].

In contrast to passively reading content, young people who actively use web-based forums—by initiating and replying to posts—can benefit from the therapeutic nature of writing and from providing support to other users in need [28]. The veil of anonymity offered by most web-based forums reduces users’ feelings of threat and inhibition when posting or responding [29,30], creating vast potential to connect young people who share similar life experiences and emotions but may not be in close geographical or physical proximity [9]. Consequently, young people may feel more motivated to post on web-based forums because they can voice their opinions with greater confidence and less fear of judgment [14]. Posting also contributes to the exchange of emotional and informational resources within the online space and encourages further engagement from users [31]. “Reply reciprocity,” whereby users tend to receive more replies to their posts if they respond to others, further underscores the importance of encouraging participation, as this positive feedback loop promotes sustained user activity [31].

Several barriers to the use of web-based mental health forums have also been identified. Concerns about confidentiality and privacy can discourage posting on these forums, as can difficulties in accurately conveying emotions through writing [22]. In addition, reading about the challenges faced by other users may be triggering and induce negative moods [32]. Young people are also deterred from reading posts when forums lack high-quality resources or relevant topics [18,22]. Fears of causing harm to oneself (eg, worsening one’s mental health condition) or to others (eg, making others feel worse) have likewise been identified as barriers to active participation [23]. Furthermore, some young people cite the lack of orientation for new users—making it harder to fit into a new online environment—as an additional barrier to active participation in mental health forums [32].

While existing studies have examined factors influencing engagement and proposed ways to enhance young people’s experiences with mental health forums, such efforts are rarely guided by a comprehensive intervention framework like the BCW. Moreover, most studies on young people’s use of web-based mental health forums take a one-dimensional view of engagement, without exploring the specific behaviors involved in interacting with these platforms. This limits researchers’ and practitioners’ ability to identify the distinct factors influencing different engagement behaviors and to develop effective, targeted solutions.

### Behavior Change Wheel Framework

**Figure 1:**
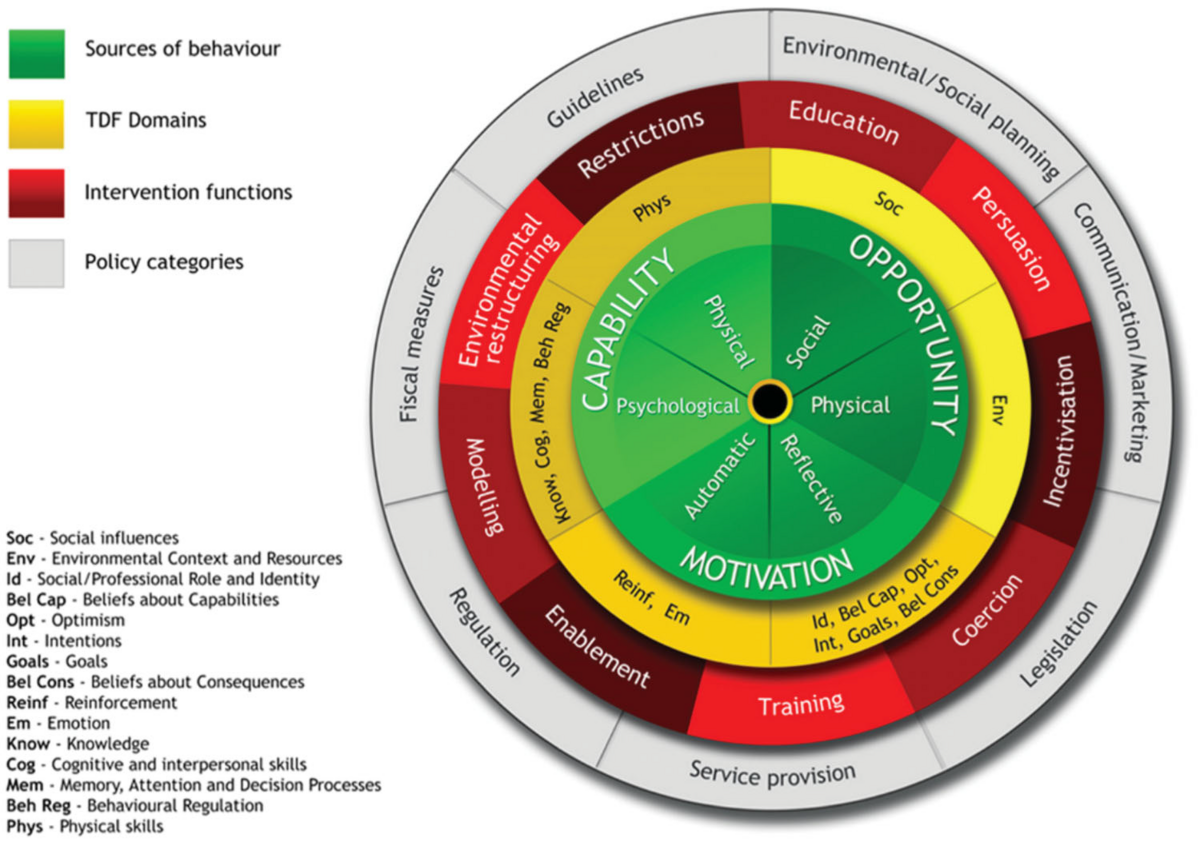
The Behavior Change Wheel and Theoretical Domains Framework (TDF).

The BCW (Figure 1) is a comprehensive framework developed through the integration of 19 existing behavior change theories [21]. It offers a structured, evidence-based approach for diagnosing the barriers and enablers of behavior, which in turn informs the design and evaluation of interventions [33]. Central to the BCW is the COM-B model, which posits that capability (physical and psychological), opportunity (social and physical), and motivation (reflective and automatic) interact to produce behavior. The Theoretical Domains Framework (TDF) is often used alongside the COM-B model to provide greater granularity [34] and a more detailed understanding of different types of behavioral influences.

Nine intervention types that enable behavior change form the layer surrounding the COM-B model, followed by an outer layer outlining 7 policy options for delivering these interventions [35]. The Theory and Techniques Tool [36] facilitates the mapping of TDF domains to behavior change techniques (BCTs) within the behavior change techniques taxonomy (BCTTv1). The taxonomy contains 93 evidence-based BCTs that are observable and replicable, facilitating behavior change [37]. This enables identified barriers to target behaviors to be linked to specific BCTs to achieve the desired change. The BCW approach has been successfully applied to enhance web-based mental health services for young people, including webchat counseling [38,39], helpline services [40], peer support groups [41], and self-harm content on a mental health forum [42].

### Current Study

This study applies the BCW framework [20] to systematically explore the perceived barriers and facilitators of engagement in UK-based web-based youth mental health forums among young people aged 16-25 years. Through qualitative interviews, this study addresses the following research questions:

- Using the COM-B/TDF, what are the barriers and enablers to young people’s engagement with youth mental health forums, specifically in relation to posting, responding, and reading?
- Using the BCTTv1, which BCTs can be applied to enhance young people’s engagement with youth mental health forums, focusing on posting, responding, and reading?

This study can inform strategies to optimize the design and delivery of youth mental health forums, helping to better harness the potential of these web-based platforms in supporting young people’s mental health and well-being.

## Methods

### Participants

The study targeted young people aged 16-25 years who had read, posted, or responded to posts in UK-based youth mental health forums. Participants were recruited via convenience sampling, with support from The Mix, powered by Mental Health Innovations—a UK-based charity that provides mental health support for young people, including crisis helplines, support groups, and web-based forums. The Mix helped publicize the study by sharing a recruitment poster and registration link via their web pages and social media channels. Additionally, the study was promoted on LinkedIn (LinkedIn Corporation) to broaden the pool of potential participants. Interested young people registered through an online form, which collected information such as age, gender, ethnicity, and frequency of youth mental health forum use.

Of the 32 young people who registered interest, 12 did not meet the eligibility criteria (eg, outside the target age range or not using youth mental health forums) and were excluded. Of the remaining 20 eligible participants, 6 did not provide consent after receiving the participant information sheet and consent form. Fourteen participants were interviewed; however, 1 interview was later excluded due to possible duplication.

The mean age of the 13 participants was 21 years, with a range of 17-25 years; 3 participants self-identified as male, 8 as female, 1 as nonbinary, and 1 did not wish to disclose their gender. Table 1 provides further details on participants’ characteristics.

**Table 1:** Description of participants’ characteristics.

Participant ID	Age (years)	Gender	Self-identified ethnicity	Experience with replying to posts on youth mental health forums	Mental health forums used
1	19	Woman	White British	Weekly	The Mix
2	25	Man	Indian	Weekly	The Mix
3	21	Woman	Iranian	Daily	The Mix
4	20	Man	Black African	Weekly	The Mix
5	23	Woman	Multiple ethnic groups	Never	The Mix
6	25	Woman	White British	Monthly or less	The Mix
7	18	Nonbinary	White British	Weekly	The Mix
8	23	Did not disclose	White British	Never	The Mix
9	23	Woman	Asian	Never	The Mix
10	23	Man	White British	Daily	The Mix and Kooth
11	24	Woman	Black African	Daily	Kooth
12	18	Woman	Black Caribbean	Monthly or less	The Mix
13	17	Woman	Multiple ethnic groups	Weekly	The Mix

### Procedure

The researchers (ZL, SYL, and SL) conducted 1-on-1 semistructured interviews with participants from June to July 2024. These interviews were held online via Microsoft Teams. Participants could choose to respond using either audio conferencing or written chat, depending on their stated preference. All participants were asked the same set of questions based on an interview guide developed using COM-B and TDF domains, along with general questions that allowed for a broader range of responses. The guide was developed by ZL, SYL, and SL in consultation with the senior author (LMG), and the questions were adapted from previous studies with a similar focus [38]. Sample questions were “What discourages you from responding to a post?” (general question), “What do you think are the benefits of using web-based mental health forums?” (reflective motivation: beliefs about consequences), and “Do others such as friends, family, or other users affect whether you use web-based mental health forums?” (social opportunity: social influences). The full interview schedule is provided in Multimedia Appendix 1.

Before recruitment began, the interview schedule was piloted with 4 young people from different demographic backgrounds (eg, gender, age range) to ensure that the questions were easily understood and that the interview could be completed within 30-45 minutes. Based on their feedback, the schedule was revised to use less technical language to enhance comprehension. The revised version also included additional prompts to encourage participants to provide more in-depth responses.

Publicity and participant recruitment began after the interview schedule was finalized. Interested individuals who met all eligibility criteria were provided with a consent form and a participant information sheet outlining the details of the study. After reviewing the information sheet and returning a signed consent form, participants were scheduled for 1-on-1 online interviews conducted by the research team. The interviews explored 3 types of behavioral engagement with web-based forums: reading forum content, creating new posts, and responding to existing posts. All interviews were recorded and transcribed. Participants received £25 (US $36) worth of vouchers as compensation for their participation.

### Data Analysis

The behaviors of posting, responding to posts, and reading posts on youth mental health forums were coded separately by ZL, SYL, and SL. For each behavior, analysis followed a deductive approach guided by the Framework Analysis method, which included the stages of transcription, data familiarization, indexing, charting, and data interpretation [43]. Transcription and data familiarization involved repeatedly reviewing the interview content to develop a deeper understanding of the data. Indexing referred to applying an existing analytical framework to the dataset, while charting involved systematically organizing the data [44]. To support the indexing and charting process, a codebook was developed to define how each TDF domain applied to the specific engagement behaviors associated with youth mental health forum use (see Multimedia Appendix 2 for an example). The TDF served as a useful framework for coding and conducting a behavioral diagnosis to identify participants’ perceived barriers and facilitators to forum use.

To enhance the reliability and consistency of coding, and to provide additional perspectives on the data, half of the total transcripts were second-coded by either ZL, SYL, or SL. Feedback from this process was used to refine the codebook applied throughout the deductive analysis phase. This was an iterative process in which the codebook was continually updated during transcript coding. The overall agreement rate exceeded 80% (122/146, 83.5%), and all coding discrepancies were discussed and resolved through mutual agreement.

After indexing and charting the data using the co-developed codebook, an inductive analysis approach was adopted to identify more specific and concrete themes. Candidate themes were examined to ensure internal consistency, and the data extracts within each theme were reviewed to confirm accurate representation, with further refinements made where necessary. Elements of Braun and Clarke’s [45] reflexive thematic analysis were incorporated, emphasizing reflective and purposeful engagement with the data informed by the researchers’ subjective experiences.

Following the deductive and inductive analyses, core inductive themes were shortlisted by ZL and LMG [46] based on their frequency and their importance and relevance to at least 2 of the 3 target behaviors. This criterion was used to identify key areas for improvement. Based on the identified TDF domains, associated BCTs with conclusive evidence-based links in the Theory and Techniques Tool were considered, while those with inconclusive or absent links were excluded. The final selection of BCTs and corresponding intervention strategies was informed by previous research on web-based forums and assessed for appropriateness and feasibility within this context by ZL, SYL, and SL. The selected strategies were further evaluated using the APEASE (Affordability, Practicability, Effectiveness, Acceptability, Side-effects, and Equity) criteria [21].

### Ethical Information

This study was approved by the UCL Research Ethics Committee (reference number 16583/003; approval date: 27 July 2023). Informed consent was obtained, with researchers highlighting that participation was voluntary and that participants’ sharing would be kept confidential.

## Results

### Overview

Ten core inductive themes were generated and mapped to the corresponding TDF domains (Table 2). These comprised 3 enablers, 2 barriers, and 5 that functioned as both enablers and barriers. Each theme is described in detail below, supported by participant quotes identified by their participant ID (Table 1). Themes specific to creating posts, responding to posts, and reading posts were also identified (see Multimedia Appendix 3).

**Table 2:** Description of core inductive themes.

Theme	Barrier/enabler	Description of theme	Relevant behaviors
Positive emotions	Enabler	Young people feel positive emotions (eg, relief, calmness) after using youth mental health forums.	Posting, responding, and reading
Beliefs about helpfulness of the reply	Barrier	Young people are discouraged from creating a new post or responding if they felt that the replies would not be helpful.	Posting and responding
Presence of moderators	Barrier/enabler	Presence of moderators on youth mental health forums makes the web space safer and reduces antisocial behavior, but may also limit the content of posts.	Posting, responding, and reading
Relevance of information	Barrier/enabler	Content that is not properly categorized or not personally relevant to young people discouraged reading and responding, but may encourage posting behavior.	Posting, responding, and reading
Accessing content on mobile phones	Barrier/enabler	The ability to access forum content on mobile phones facilitates usage, but when the layout is not optimized, it leads to difficulty in typing and reading on the go.	Posting, responding, and reading
Anonymity	Enabler	Anonymity reduces the sense of judgment from other users and encourages the sharing of thoughts.	Posting and responding
Time taken to obtain support	Barrier/enabler	Being able to quickly find the information needed can encourage young people to read posts, while the slow support and response prevent them from posting new content.	Posting and reading
Interactions with other users	Barrier/enabler	Positive experiences with other users facilitate posting and responding, while negative interactions discourage young people from doing so.	Posting and responding
Empathy	Enabler	Empathy helps young people relate to the original poster and provide more respectful and effective responses.	Responding and reading
Poor communication skills	Barrier	Poor communication skills hindered young people from creating and responding to posts in youth mental health forums. This barrier also had implications for reading, making it harder to understand posts.	Posting, responding, and reading

### Automatic Motivation (TDF: Emotions)—Positive Emotions (Enabler)

Experiencing positive emotions was identified as an enabler for half of the young people using youth mental health forums across posting, responding, and reading behaviors. For those who actively engaged by posting or responding, these emotions often arose from the sense of connection with others. For example, P12 shared: “I feel relieved, I feel I'm able to interact (with) someone, I'm able to at least remedy a situation for someone, and I feel satisfied (with) myself.” P10 expressed similar sentiments, stating that they felt “a relief and calmness within that you've actually add(ed) value to someone’s life”. Even participants who only read forum posts reported experiencing positive emotions, often in the form of validation for their thoughts or behaviors. For example, P1 described: “it’s nice to read the posts and see that I’m not the only one feeling a certain way or experiencing a specific thing.”

### Reflective Motivation (TDF: Beliefs About Consequences)—Beliefs About Helpfulness of the Reply (Barrier)

A barrier that discouraged a small number of young people from posting or responding was their belief about the helpfulness of replies. For example, P6 noted that responses are “not always helpful, depending on who responds.” P8 expressed a similar concern, highlighting that users may not “(get) the answers you wanted.” Specifically for the behavior of responding to posts, P1 reported sometimes withholding their response, explaining that they “might say the wrong thing to the person and end up making them feel worse or leave them feeling more confused.” Others expressed similar concerns, noting that they were cautious about responding to “avoid upsetting or triggering other users” [P1], or to ensure they did not “make things worse for anyone” [P6].

### Physical Opportunity (TDF: Environmental Context and Resources)

#### Presence of Moderators (Barrier/Enabler)

For most young people, the presence of moderators in the forums could act as either a barrier or an enabler to posting, responding, and reading posts. Some participants noted that moderators helped make the online space safer, which in turn encouraged forum engagement. As P4 explained, “professional moderation on the online environment can ensure that the discussion boards remain (a) safe and respect(ful) space.” Participants also highlighted that moderators helped prevent exposure to content they preferred not to see, such as “seeing feeds that you think you don’t want to associate with or relate with, or even see” [P11], including “sexually explicit posts, political posts, terrorism, crimes” [P8]. Conversely, a small number of young people felt that moderators could limit the content of posts and restrict self-expression. As P13 noted, “you’re not able to adequately share what you're thinking about...the moderators put a lot of limitations that are making you not maybe express yourself the way you would have done if the moderator had no restrictions.”

#### Relevance of Information (Barrier/Enabler)

A small group of young people noted that the relevance of information could act as either a barrier or an enabler for engagement, including posting, responding, and reading. For example, P6 explained, “there are a lot of subsections which...is needed but sometimes posts are in the wrong area or there are so many that things get lost” [P6]. Some young people also reported that the content they encountered on the forums was “not always relevant” and often focused on the “popular ones” [P3]. This perception that discussion board information might not be personally relevant or useful sometimes discouraged them from reading posts. As P3 explained, “whenever I read stuff about other people there, their situation isn’t like mine.” These participants therefore preferred creating posts on the forums rather than searching for information in existing posts, as posting could elicit responses that were more personally relevant and “felt more tailored to me and my current situation” [P6]. However, the inability to find relevant information also acted as a barrier, limiting young people’s capacity to share knowledge and provide useful responses. P8 also noted that the presence of posts that were “repetitive or non-unique” discouraged her from responding and added that she “would respond to posts more (if) they were more varied.”

#### Accessing Content on Mobile Phone (Barrier/Enabler)

For a small number of young people, accessing forum content on mobile phones could function as either an enabler or a barrier for posting, responding, and reading behaviors. One participant highlighted that the convenience of using her phone facilitated forum engagement: “the fact I can use it both on my phone and my laptop make me use it more because I can access it whenever I want” [P1]. However, others reported that the website’s design and mobile experience were not optimized, which hindered their ability to use the forum on the go. As P5 elaborated, “If you’re checking in via mobile phone, it can be a little awkward to navigate, some areas oversized or needing to be zoomed in upon, not always straightforward to type something in.” Others shared similar challenges, with P9 stating that “using the website on cellphone is not convenient...It could be better if it launch apps,” while P1 suggested that a mobile application could be helpful, allowing users to “access it easier” and “get notifications.”

#### Anonymity (Enabler)

In the interviews, most young people highlighted anonymity as a key factor facilitating active engagement in forums, particularly for posting and responding. A secure, anonymous, and private environment was considered crucial for users to feel comfortable sharing their thoughts and concerns, thereby encouraging young people to create posts to seek help. For example, “I think the biggest contributor to me using it is the fact that it’s anonymous so I don't have to worry about people I know seeing my posts” [P1] and “the sense of anonymity to most people so it’s easier to open up about something and get the support” [P6]. When crafting responses, the sense of anonymity enabled young people to “freely talk about your situation or feelings without anyone judging you or questioning or invalidating you” [P1] and to “open up without the fear of people knowing who you are” [P11]. Anonymity facilitated engagement by allowing participants to share their thoughts with reduced concern about judgment from others.

#### Time Taken to Obtain Support (Barrier/Enabler)

For a small group of participants, the time required to obtain support could act as either an enabler or a barrier to posting and reading, depending on how quickly responses were received. Some participants preferred reading existing posts rather than creating new ones to seek information, as the immediacy of support available through reading was more appealing in the context of web-based forums. The wealth of information in the existing forum posts led some participants to view reading posts as a more desirable alternative to creating new posts. As P12 explained, “once I log in, I notice that most of the information that I needed to post, was already sometimes already there.” This view was echoed by P8, who noted, “generally making posts means you can be waiting a couple of days for a response, whereas when you search and read information you get what you need immediately.” The immediacy of support thus encouraged participants to read posts, while the potential for delayed responses discouraged them from posting.

### Social Opportunity (TDF: Social Influences)—Interactions With Other Users (Barrier/Enabler)

For a small group of young people, interactions with other forum users specifically influenced active engagement (ie, posting and responding), acting as either an enabler or a barrier depending on the nature of these interactions. Participants who had positive experiences were more likely to increase their posting or responding behaviors, whereas those with negative experiences were less inclined to engage. For example, P6 described the role of reciprocity in deciding whether to reply, stating, “if someone has supported me before and they post I would be more likely to reply...It just feels like you are then helping someone who has helped you before.” Conversely, negative experiences with other users discouraged young people from responding. As P11 explained, “usually if its someone that I know doesn’t like me I will never reply to them...if there have been issues in the past with people it makes you want to avoid them.” The impact of negative interactions extended beyond specific users to affect overall posting and responding behaviors. For example, “negative comments or replies by others” [P7] or instances where others “answer(ed) you rudely...really discourage(d)” [P13] deterred users from posting in general, whereas experiences of “peer support and empathy and understanding” [P4] encouraged engagement.

### Psychological Capability (TDF: Skills)

#### Empathy (Enabler)

For most young people, empathy was identified as an important skill that helped them relate to the original poster when reading posts and providing responses. Specifically, having empathy helped users “understand the situation...(and) how they’re feeling” [P2], facilitating “emotional connection” and “driv(ing) you to offer support” [P4]. It also provided a foundation for young people to reply “respectfully and in an understanding way” [P6]. P11 elaborated that a lack of empathy could lead to negative outcomes: “they may tend to just reply without any emotions and for the person that has gone through this, it’s hurtful and this discourages them from posting something that they feel like it’s very personal or made them feel like that.” P1 echoed these points, noting that empathy is necessary to “fully understand what that person is feeling before you begin to reply...otherwise the person may end up feeling more isolated or misunderstood.”

#### Poor Communication Skills (Barrier)

Half of the participants reported that the poor communication skills of posters and responders discouraged them from engaging in posting and responding on youth mental health forums. For example, P3 stated, “I don’t have great communication skills...that’s why I don’t always tend to write.” P4 added that having good writing skills helped to “express your ideas and what you are going through.” This barrier also affected the behavior of reading, representing a Physical Opportunity (TDF: Environmental Context and Resources) barrier, as young people were deterred from reading posts that were poorly communicated. This included posts that lacked clarity and coherence, such as those with incorrect grammar and punctuation, which made them more difficult for participants to read and understand. P13 explained, “if you’re reading through a post that has grammatical mistakes...you can interpret it differently, but if it is clear, then you just get the information the way it is.” In addition, some posts were described as “vague” [P9] or “confusing” [P10], with P9 adding that “some of the posts didn’t make much sense...even after reading them.”

### BCTs

To address the identified barriers, the Theory and Techniques Tool was used to identify relevant BCTs. The most appropriate intervention components were then selected based on an APEASE assessment, as detailed in Table 3.

**Table 3:** Proposed intervention components and APEASE^a^ evaluation.

Barrier (TDF^b^ domain)	Suggested BCT^c^	Description	APEASE evaluation
Presence of moderators (Environmental Context and Resources)	Adding objects to the environment Restructuring the physical environment	Clearly state rules and guidelines about content that is not permitted, and provide different interaction mechanisms for users	Creating clear guidelines is an affordable, acceptable, and practicable way of setting the boundaries of what can be discussed in web-based forums. Having different interaction mechanisms can also be effective in providing the required social support for young people with different support needs [47].
Relevance of information (Environmental Context and Resources)	Restructuring the physical environment	Implement recommender engines (ie, tools which use machine learning to recommend relevant items to users) to improve the recommendation of relevant content	While it may not be the most affordable option, it is the most effective, practicable, and acceptable in addressing stated barrier [48]. Thus, organizations are suggested to consider this option if resources permit.
Accessing content on a mobile phone (Environmental Context and Resources)	Restructuring the physical environment	Optimize website layout on mobile interface Develop a mobile app	An effective, practicable, and acceptable option to improve the readability and usability of forums on mobile devices. [49] Effective and acceptable by young people, but may be less affordable than optimizing layout on the mobile interface [50]. Organizations may wish to consider this if resources permit.
Time taken to obtain support (Environmental Context and Resources)	Restructuring the physical environment	Leverage technology to facilitate quicker responses (eg, using automated triage systems; artificial intelligence as virtual assistants to help with replies) Young people could be involved in developing the artificial intelligence tool to ensure it is acceptable, effective, and inclusive.	Effective and acceptable, having the potential positive side effect of reducing the workload of moderators [51,52]. However, it is less affordable than having manual processes in place to prioritize replies, and organizations may wish to consider this if resources permit.
Interactions with other users (Social Influences)	Social reward Social support (unspecified)	Moderators validate young people who create their first post or respond to posts; badges Unmoderated forums to introduce moderators who can actively look out for negative interactions	This is an affordable, practicable, and acceptable way to increase positive interactions on the forum, as such social interactions could improve mood and increase intrinsic motivation [53]. For forums without moderators, this is an effective, practicable, and acceptable way to manage negative interactions between users [9,28].
Poor communication skills; self-understanding of emotions (Skills)	Instruction on how to perform behavior	Online self-administered training on ways to communicate effectively; self-paced learning tools/modules	This option of using training to improve young people’s communication skills is practicable [54]. An online self-administered training was assessed to be more effective than other methods of instruction, such as email (not suitable as it may be too wordy and hard for young people to absorb the information) or in-person training (more resource-intensive and difficult to accommodate available timeslots).
Beliefs about helpfulness of reply (Beliefs About Consequences)	Information about social and environmental consequences	Customized email outreach highlighting the positive consequences of posting and replying to forum posts	This is an affordable and practicable option to convey succinct information. Using a personalized and persuasive tone in these types of communications has been shown to be effective in increasing participation in web-based communities [55].

^a^APEASE: Affordability, Practicability, Effectiveness, Acceptability, Side-effects, and Equity.

^b^TDF: Theoretical Domains Framework.

^c^BCT: behavior change technique.

## Discussion

### Principal Findings

This study addresses an existing gap by examining the factors influencing young people’s engagement in youth mental health forums, including creating posts, responding to posts, and reading posts. Ten core inductive themes were identified across 5 TDF domains, including Skills, Beliefs About Consequences, Emotions, and the Social Influences and Environmental Context and Resources. Of these, 3 themes were enablers, 2 were barriers, and 5 functioned as both enablers and barriers. Some themes were relevant to all 3 behaviors, such as the presence of moderators, while others were specific to more active participation (ie, posting and responding), such as positive interactions with other users. While most themes were relevant to either active participation or overall engagement, some acted as a barrier for 1 behavior but an enabler for another, or were specific to responding and reading only. For example, the ability to quickly find the information needed encouraged reading posts, whereas slow support and responses acted as a barrier to posting new content. Empathic skills were important for both reading and responding. Most barriers were related to physical and social opportunity, suggesting that adjustments to the service structure could enhance young people’s engagement. Below, the enablers of engagement are discussed first, followed by the barriers, along with the proposed contextualized BCTs to optimize engagement.

### Enablers of Engagement With Youth Mental Health Forums

A range of capability, opportunity, and motivation factors influence young people’s use of youth mental health forums. Regarding automatic motivation, this study found that both passive and active engagement with the forum can generate positive feelings, which serve as reinforcement to encourage similar behaviors in the future. This aligns with Smith-Merry et al’s [28] findings, which reported that users of web-based mental health forums viewed responding as therapeutic and associated with positive emotions.

Regarding psychological capability, many young people in the study highlighted empathy as an important skill for responding to and reading others’ posts. Empathy enables users to better understand others’ situations and provide sensitive, thoughtful replies. Perowne and Gutman’s [42] study found that empathy in communication was a key skill for moderators of self-harm content on young people’s web-based forums, and this same skill is required by users to effectively understand and respond to others’ posts.

Regarding physical opportunity, young people in this study reported that a platform allowing anonymity enabled them to express their opinions with less fear of judgment, thereby facilitating posting and responding behaviors. This finding aligns with research in other fields, such as education and physical health, which indicates that anonymity generally promotes self-disclosure and facilitates engagement with web-based forums [56-58].

### Barriers to Engagement and Suggested BCTs

#### Presence of Moderators

Previous studies [9,59] have demonstrated that forum moderators play a crucial role in maintaining a safe online environment for participation. This was echoed in our study, where participants reported that the presence of moderators facilitated engagement by mitigating potential adverse effects of anonymity, such as bullying or disrespectful behavior resulting from disinhibition [14]. This study extends existing literature by highlighting a potential drawback of moderation: some young people may be reluctant to post for fear of negative consequences, such as being banned, if they freely express their thoughts or experiences. The BCTs of “adding objects to the environment” and “restructuring the physical environment” may help mitigate this issue. Specifically, forum providers can reduce ambiguity by offering clear rules or guidelines regarding content that should not be posted [60], giving young people greater certainty about what is acceptable on the forums. Additionally, alternative interaction mechanisms (eg, 1-on-1 chats, if available) can be offered for users to share personal anecdotes of a sensitive nature, ensuring they continue to feel supported for their mental health needs [47]. Providing such avenues allows young people to express their thoughts safely while reducing the likelihood of posting inappropriate content on web-based forums.

#### Relevance of Information

Consistent with past studies [29], another barrier to reading and responding to posts in youth mental health forums was the difficulty in finding relevant information. Most forums organize posts by recency (ie, the most recent posts appear at the top) rather than providing customized content tailored to users’ interests. Furthermore, when forum content is not categorized intuitively or specifically enough (eg, a category is too generic and includes posts spanning a wide range of sub-topics), it hinders young people’s ability to find personally relevant information. This, in turn, affects their ability to read or share knowledge and reduces their confidence in replying effectively. Interestingly, the inability to find relevant information may actually increase posting behavior, as users attempt to obtain content that is more personally relevant. However, when young people post solely because they cannot easily locate existing information, it can contribute to more repetitive content on the forums. Thus, it is important to optimize content relevance to reduce repetitive information and maintain the quality of interactions in the forums. To help young people find information that is relevant for reading and responding, forum providers could apply the BCT of “restructuring the physical environment,” delivering more relevant or customized content based on users’ previously indicated preferences. Implementing recommender engines based on participant preferences has been shown to enhance forum engagement, and this approach could be applied in the current context, as young people are more likely to read and respond to posts they find personally relevant [48].

#### Accessing Content on a Mobile Phone

Many young people value the convenience, immediacy, and ease of accessing services via their mobile phones [61]. Consequently, difficulties in using web-based forums on mobile devices—such as challenges with reading or typing—generally hindered posting, reading, and responding behaviors. By applying the BCT of “restructuring the physical environment,” providers of youth mental health forums could optimize the layout of their websites for mobile interfaces. Additionally, forum providers might consider developing a mobile app to further facilitate ease of use on mobile devices. Converting websites into mobile apps offers several advantages, including the ability to provide instantaneous notifications (if users allow) and to sort content based on user preferences [50]. Providing more customized content through mobile apps could also help address the barrier of difficulty in finding relevant information on the forums. This, in turn, may lead to higher engagement and increased responding behavior.

#### Time Taken to Obtain Support

Efficiency is a key factor influencing user engagement in help-seeking and information-seeking via web-based forums [62]. Studies by Browne et al [63] and Lobban et al [12] found that forum users valued timely responses to their posts, as this helped them address their current difficulties. Similarly, users who read posts to seek help may be motivated by the desire for immediate support. To minimize the time required to obtain assistance on web-based forums, providers could apply the BCT of “restructuring the physical environment,” leveraging technology to facilitate faster responses. For example, previous studies have highlighted the utility of automated triage systems to help moderators identify posts requiring immediate attention [51], as well as the use of artificial intelligence as virtual assistants to respond during off-peak hours [52]. These technological interventions can reduce reliance on manual post monitoring and increase the efficiency of responses.

#### Interactions With Other Users

Consistent with previous studies [14], some young people reported reservations about posting or responding due to prior negative interactions with other users. By contrast, others noted that they were more inclined to respond to users who had previously replied to them, supporting the concept of reply reciprocity [31,64]. This factor specifically affects active engagement (ie, posting and responding) and underscores the importance of fostering positive interactions on the forums to create a reinforcing feedback loop. To encourage greater posting and responding, forum providers could apply the “social reward” BCT, with moderators placing added emphasis on recognizing users who have made their first post or responded to others, thereby motivating continued engagement. Applying the BCT of “social support (unspecified),” it is important for unmoderated youth mental health forums to introduce moderators. These moderators should actively monitor the forums for negative interactions and work to diffuse and resolve conflicts efficiently [9,28]. By increasing positive interactions and reducing negative ones, overall active engagement with youth mental health forums can be facilitated.

#### Poor Communication Skills

Existing literature highlights communication skills as a key factor influencing forum engagement. Poor communication skills can act as a barrier, limiting young people’s ability to post about their mental health issues or respond to others’ posts. Consequently, young people had difficulty understanding poorly written posts, which hindered their reading. Previous research also indicates that longer posts can impede reading, as users are more likely to lose focus unintentionally [65]. Unclear or incoherent posts and replies can also affect users’ ability to understand and act on the information provided [66,67]. To enhance communication skills, youth mental health forum providers could implement the “instruction on how to perform behavior” BCT. This might involve an online, self-administered training module that young people can access at their convenience. The training could include guidance on posting and replying effectively (eg, being clear and concise) and emphasize the importance of responding to other users’ replies when they agree with the content. Although not in the same context, previous research has shown that online, self-administered social skills training can effectively reduce social fear and enhance social skills [54]. This highlights the potential of using online, self-administered training to enhance young people’s skills and confidence in contributing positively to forums.

#### Beliefs About Helpfulness of Reply

A recent systematic review indicated that perceived effectiveness of professional help is a key factor influencing mental health help-seeking behavior. Adolescents’ beliefs about the helpfulness of advice or interventions can significantly affect their likelihood of seeking support; for example, they may avoid seeking help if they have doubts about its effectiveness [68]. An empirical study of a web-based mental health community intervention also found that the quality and effectiveness of responses influenced young people’s behavior in creating posts to seek help online [69]. To address the barrier of negative perceptions regarding the helpfulness of replies, forum providers could consider making the benefits of using web-based mental health forums more salient to users. The BCT “information about social and environmental consequences” could be used to emphasize the positive outcomes of posting or replying to forum posts. This could be paired with an active call to action, encouraging users to create posts or respond to existing ones. Implementation could include customized email outreach to new forum members or to existing members who have not yet posted or responded. The email could highlight the positive outcomes of posting or responding helpfully to other users, including validating those who have replied to the original post. It could also encourage recipients to complete the online, self-administered training mentioned above, helping to build their confidence in posting or replying. Directly reaching out to forum members with a persuasive message can effectively promote responding behavior. In similar contexts, studies have shown that actively inviting users to participate in discussions on web-based forums leads to increased engagement [55].

### Limitations

The following limitations should be considered when interpreting the findings of this study. First, the use of convenience sampling may have introduced self-selection bias, meaning that individuals who consented to participate in the interviews could differ systematically from those who did not [70]. A similar study by Prescott et al [29] found that participants who volunteered for interviews were more likely to emphasize the advantages of web-based mental health forums rather than the disadvantages, possibly because individuals with more positive experiences were more inclined to share their opinions. To mitigate this, participants were assured of the anonymity of their responses and encouraged to provide honest feedback. Future research could also aim for a more representative sample, including a balanced mix of genders, as there may be gender differences in the use of online platforms for mental health support [8]. Additionally, most participants in this study used a single web-based mental health forum in the United Kingdom, which may limit the generalizability of the findings to other forums in different contexts. Future research could explore the barriers and enablers to posting, responding, and reading posts on other mental health forums targeting different age groups or populations. As the proposed intervention strategies were not evaluated in this study, future research could assess their effectiveness in optimizing young people’s engagement with web-based mental health forums. Finally, as forums represent only a subset of the online mental health ecosystem, it may be valuable to explore how these strategies could be applied more broadly to other platforms, such as social media, to maximize their impact.

### Conclusions

In today’s digitally connected age, the internet serves as a valuable conduit for knowledge sharing and building connections. Youth mental health forums have the potential to provide accessible support, complement in-person services, and positively contribute to young people’s mental well-being. However, a lack of engagement can limit the impact and effectiveness of these forums in supporting young people. This study contributes to the existing knowledge by examining the various ways in which young people engage with youth mental health forums. Different strategies may be prioritized depending on whether forum providers aim to increase active engagement (eg, encouraging posting and responding through positive interactions) or overall engagement (eg, implementing moderators and clear rules to support both active and passive participation).

Overall, these findings demonstrate the utility of the BCW approach in conducting behavioral diagnoses and developing behavior change interventions, contributing to the evidence supporting the application of behavior change frameworks in digital mental health interventions. This can help advance the emerging field of digital mental health intervention engagement, ultimately leading to higher-quality forum services that provide better support for young people.

## Appendix

Multimedia Appendix 1 Interview schedule.

Multimedia Appendix 2 Codebook.

Multimedia Appendix 3 Unique themes for each type of engagement behavior.

## Abbreviations

APEASE: Affordability, Practicability, Effectiveness, Acceptability, Side-effects, and Equity
BCT: behavior change technique
BCTT: behavior change techniques taxonomy
BCW: Behavior Change Wheel
COM-B: capability, opportunity, and motivation—behavior
TDF: Theoretical Domains Framework

## References

[1] Ford K, Freund R (2022). Young lives under pressure: protecting and promoting young people’s mental health at a time of global crises. Young Lives.

[2] Marchant N, Masiga J How to tackle the mental health crisis facing young people. World Economic Forum.

[3] Montero-Marin J, Hinze V, Mansfield K, Slaghekke Y, Blakemore S, Byford S, Dalgleish T, Greenberg MT, Viner RM, Ukoumunne OC, Ford T, Kuyken W, MYRIAD Team (2023). Young people's mental health changes, risk, and resilience during the COVID-19 pandemic. JAMA Netw Open.

[4] McCurdy C, Murphy L We've only just begun. Resolution Foundation.

[5] Crenna-Jennings W Non-specialist mental health support for young people in England. Education Policy Institute.

[6] (2024). Children’s mental health services 2022-23. Children's Commissioner.

[7] Evans S (2017). The challenge and potential of the digital age. Transactional Analysis Journal.

[8] Faverio M, Anderson M, Park E (2025). Teens, social media and mental health. Pew Research Center.

[9] Hanley T, Prescott J, Gomez KU (2019). A systematic review exploring how young people use online forums for support around mental health issues. J Ment Health.

[10] Barak A, Dolev-Cohen M (2007). Does activity level in online support groups for distressed adolescents determine emotional relief. Couns and Psychother Res.

[11] Horgan A, McCarthy G, Sweeney J (2013). An evaluation of an online peer support forum for university students with depressive symptoms. Arch Psychiatr Nurs.

[12] Lobban F, Akers N, Appelbe D, Chapman L, Collinge L, Dodd S, Flowers S, Hollingsworth B, Johnson S, Jones SH, Mateus C, Mezes B, Murray E, Panagaki K, Rainford N, Robinson H, Rosala-Hallas A, Sellwood W, Walker A, Williamson P (2020). Clinical effectiveness of a web-based peer-supported self-management intervention for relatives of people with psychosis or bipolar (REACT): online, observer-blind, randomised controlled superiority trial. BMC Psychiatry.

[13] van Mierlo Trevor (2014). The 1% rule in four digital health social networks: an observational study. J Med Internet Res.

[14] Marshall P, Booth M, Coole M, Fothergill L, Glossop Z, Haines J, Harding A, Johnston R, Jones S, Lodge C, Machin K, Meacock R, Nielson K, Puddephatt J, Rakic T, Rayson P, Robinson H, Rycroft-Malone J, Shryane N, Swithenbank Z, Wise S, Lobban F (2024). Understanding the impacts of online mental health peer support forums: realist synthesis. JMIR Ment Health.

[15] Preece J, Nonnecke B, Andrews D (2004). The top five reasons for lurking: improving community experiences for everyone. Computers in Human Behavior.

[16] Leshed G (2005). Posters, lurkers, and in between: a multidimensional model of online community participation patterns. Cornell University.

[17] Himelboim I, Gleave E, Smith M (2009). Discussion catalysts in online political discussions: content importers and conversation starters. J Comput Mediat Commun.

[18] Prescott J, Hanley T, Ujhelyi K (2017). Peer communication in online mental health forums for young people: directional and nondirectional support. JMIR Ment Health.

[19] Banwell E, Hanley T, De Ossorno Garcia S, Mindel C, Kayll T, Sefi A (2022). The helpfulness of web-based mental health and well-being forums for providing peer support for young people: cross-sectional exploration. JMIR Form Res.

[20] Koh J, Kim Y, Butler B, Bock G (2007). Encouraging participation in virtual communities. Commun ACM.

[21] Michie S, Atkins L, West R (2014). The Behaviour Change Wheel: A Guide to Designing Interventions.

[22] Chan JK, Farrer LM, Gulliver A, Bennett K, Griffiths KM (2016). University students' views on the perceived benefits and drawbacks of seeking help for mental health problems on the internet: a qualitative study. JMIR Hum Factors.

[23] Breuer L, Barker C (2015). Online support groups for depression. Sage Open.

[24] Bond CS, Ahmed OH (2016). Can I help you? Information sharing in online discussion forums by people living with a long-term condition. J Innov Health Inform.

[25] Malik S, Coulson NS (2011). The therapeutic potential of the internet: exploring self-help processes in an internet forum for young people with inflammatory bowel disease. Gastroenterol Nurs.

[26] Liao Siling J, Truss K, Philips L, Eastwood O, Bendall S (2021). Young people's journeys of recovery from trauma: a qualitative study of narratives from internet forums. Psychol Trauma.

[27] Smit D, Vrijsen JN, Groeneweg B, Vellinga-Dings A, Peelen J, Spijker J (2021). A newly developed online peer support community for depression (Depression Connect): qualitative study. J Med Internet Res.

[28] Smith-Merry J, Goggin G, Campbell A, McKenzie K, Ridout B, Baylosis C (2019). Social connection and online engagement: insights from interviews with users of a mental health online forum. JMIR Ment Health.

[29] Prescott J, Hanley T, Ujhelyi Gomez K (2019). Why do young people use online forums for mental health and emotional support? Benefits and challenges. British Journal of Guidance & Counselling.

[30] Bargh JA, McKenna KYA (2004). The internet and social life. Annu Rev Psychol.

[31] Pan W, Shen C, Feng B (2017). You get what you give: understanding reply reciprocity and social capital in online health support forums. J Health Commun.

[32] Smithson J, Sharkey S, Hewis E, Jones RB, Emmens T, Ford T, Owens C (2011). Membership and boundary maintenance on an online self-harm forum. Qual Health Res.

[33] Michie S, West R (2013). Behaviour change theory and evidence: a presentation to government. Health Psychology Review.

[34] Atkins L, Francis J, Islam R, O'Connor Denise, Patey A, Ivers N, Foy R, Duncan EM, Colquhoun H, Grimshaw JM, Lawton R, Michie S (2017). A guide to using the theoretical domains framework of behaviour change to investigate implementation problems. Implement Sci.

[35] Michie S, van Stralen Maartje M, West R (2011). The behaviour change wheel: a new method for characterising and designing behaviour change interventions. Implement Sci.

[36] (2024). Theory and Technique Tool.

[37] Michie S, Richardson M, Johnston M, Abraham C, Francis J, Hardeman W, Eccles MP, Cane J, Wood CE (2013). The behavior change technique taxonomy (v1) of 93 hierarchically clustered techniques: building an international consensus for the reporting of behavior change interventions. Ann Behav Med.

[38] Richiello MG, Mawdsley G, Gutman LM (2021). Using the Behaviour Change Wheel to identify barriers and enablers to the delivery of webchat counselling for young people. Couns and Psychother Res.

[39] Mawdsley G, Richiello M, Gutman LM (2022). Barriers and facilitators of young people’s engagement with webchat counselling: a qualitative analysis informed by the Behaviour Change Wheel. Couns and Psychother Res.

[40] Campagnola M, Burlibasa A, Gutman LM (2022). Barriers and enablers to the delivery of email communication for a helpline service for young people. PEC Innov.

[41] Ananya A, Tuuli J, Perowne R, Gutman LM (2025). Barriers and facilitators to user engagement and moderation for web-based peer support among young people: qualitative study using the behavior change wheel framework. JMIR Hum Factors.

[42] Perowne R, Gutman LM (2024). Barriers and enablers to the moderation of self-harm content for a young person's online forum. J Ment Health.

[43] Gale NK, Heath G, Cameron E, Rashid S, Redwood S (2013). Using the framework method for the analysis of qualitative data in multi-disciplinary health research. BMC Med Res Methodol.

[44] Goldsmith L (2021). Using framework analysis in applied qualitative research. TQR.

[45] Braun V, Clarke V (2020). One size fits all? What counts as quality practice in (reflexive) thematic analysis?. Qualitative Research in Psychology.

[46] Braun V, Clarke V (2008). Using thematic analysis in psychology. Qualitative Research in Psychology.

[47] Saha K, Ernala S, Dutta S, Sharma E, De CM (2020). Understanding moderation in online mental health communities.

[48] Albatayneh N, Ghauth K, Chua F (2018). Utilizing learners' negative ratings in semantic content-based recommender system for e-learning forum. Educ Technol Soc.

[49] Radilova M, Kamencay P, Matuska S, Benco M, Hudec R (2020). Tool for optimizing webpages on a mobile phone.

[50] Jovenn C, Subaramaniam K, Jalil A (2019). The development of a forum mobile application for students.

[51] Milne DN, McCabe KL, Calvo RA (2019). Improving moderator responsiveness in online peer support through automated triage. J Med Internet Res.

[52] Liu X, Pankiewicz M, Gupta T, Huang Z, Baker RS (2025). A step towards adaptive online learning: exploring the role of GPT as virtual teaching assistants in online education. Open Science Framework.

[53] Kraut R, Resnick P, Kiesler S (2012). Building Successful Online Communities: Evidence-Based Social Design.

[54] Lehenbauer M, Kothgassner OD, Kryspin-Exner I, Stetina BU (2013). An online self-administered social skills training for young adults: results from a pilot study. Computers & Education.

[55] Harper F, Frankowski D, Drenner S (2007). Talk amongst yourselves: inviting users to participate in online conversations.

[56] Wadden D, August T, Li Q, Althoff T (2021). The effect of moderation on online mental health conversations.

[57] Roberts LD, Rajah-Kanagasabai CJ (2013). "I'd be so much more comfortable posting anonymously": identified versus anonymous participation in student discussion boards. AJET.

[58] Jones R, Sharkey S, Ford T, Emmens T, Hewis E, Smithson J, Sheaves B, Owens C (2018). Online discussion forums for young people who self-harm: user views. Psychiatrist.

[59] Kendal S, Kirk S, Elvey R, Catchpole R, Pryjmachuk S (2017). How a moderated online discussion forum facilitates support for young people with eating disorders. Health Expect.

[60] Mokkenstorm Jan K, Mérelle Saskia Y M, Smit JH, Beekman ATF, Kerkhof AJFM, Huisman A, Gilissen R (2020). Exploration of benefits and potential harmful effects of an online forum for visitors to the suicide prevention platform in the Netherlands. Crisis.

[61] O'Dea B, Han J, Batterham PJ, Achilles MR, Calear AL, Werner-Seidler A, Parker B, Shand F, Christensen H (2020). A randomised controlled trial of a relationship-focussed mobile phone application for improving adolescents' mental health. J Child Psychol Psychiatry.

[62] Kruzan KP, Fitzsimmons-Craft EE, Dobias M, Schleider JL, Pratap A (2022). Developing, deploying, and evaluating digital mental health interventions in spaces of online help- and information-seeking. Procedia Comput Sci.

[63] Browne NL, Carragher NO, O’Toole A, Pimm J, Ryder J, Thew GR (2022). Evaluating user experiences of SHaRON: an online CBT-based peer support platform. tCBT.

[64] Chang PF, Whitlock J, Bazarova NN (2018). “To respond or not to respond, that is the question”: the decision-making process of providing social support to distressed posters on Facebook. Social Media + Society.

[65] Forrin ND, Mills C, D’Mello SK, Risko EF, Smilek D, Seli P (2020). TL;DR: longer sections of text increase rates of unintentional mind-wandering. The Journal of Experimental Education.

[66] Berry N, Lobban F, Bucci S (2019). A qualitative exploration of service user views about using digital health interventions for self-management in severe mental health problems. BMC Psychiatry.

[67] Murnane EL, Cosley D, Chang P, Guha S, Frank E, Gay G, Matthews M (2016). Self-monitoring practices, attitudes, and needs of individuals with bipolar disorder: implications for the design of technologies to manage mental health. J Am Med Inform Assoc.

[68] Radez J, Reardon T, Creswell C, Lawrence PJ, Evdoka-Burton G, Waite P (2021). Why do children and adolescents (not) seek and access professional help for their mental health problems? A systematic review of quantitative and qualitative studies. Eur Child Adolesc Psychiatry.

[69] Li J, Liu D, Wan C, Liang Z, Zhu T (2023). Empirical study of factors that influence the perceived usefulness of online mental health community members. Psych J.

[70] Costigan CL, Cox MJ (2001). Fathers' participation in family research: is there a self-selection bias?. Journal of Family Psychology.

