# Supplementary material for: Barriers and Enablers to Young People’s Posting, Responding, and Reading Behaviors on Mental Health Forums Using the Behavior Change Wheel: Qualitative Study
Source: JMIR Hum Factors. 2025 Oct 31;12:e71549. doi: 10.2196/71549 (PMC12619017; doi:10.2196/71549)
Supplement: Multimedia Appendix 1 [file humanfactors_v12i1e71549_app1.docx]

Introduction:

Hello, and thanks for meeting with me today. I'm [*Name*], a Masters student from UCL, and I’m doing research about young people’s use of discussion boards. There are three ways people use discussion boards: (i) Searching and reading content (i.e., just browsing), (ii) Starting a new post/thread, or (iii) Replying to posts. As part of our research, we want to find out what influences the way you use discussion boards.

Before we start, I want to remind you that your participation is voluntary, and everything you say will be kept confidential. If you have any questions about the study, please let me know. I'll also be taking notes and recording our conversation. Is that okay with you?

Let's begin. I understand that you have [*read, posted and/or replied to posts*] and do this roughly once every [*frequency of interaction e.g. week, month*]. I will be asking some questions related to your usage of discussion boards.

Questions:

1. Can you briefly tell me about how you use the discussion boards?
   1. When you encounter problems or have a question, do you prefer to search and read information in the discussion boards or create new posts to seek help/advice? *[Ask participant to elaborate on their answer / explain their preferences]*
   2. Why do you read posts on discussion boards? Do you think the information is helpful? Is it easy to search for relevant information? How so?
2. Are there any features that you are not sure about how to use? (e.g., starting a new discussion thread, responding or reacting to existing comments) Could you give an example?
3. What encourages you to create a post?
4. What prevents/discourages you from creating a post
5. What encourages you to respond to a post?
6. What prevents/discourages you from responding to a post?
7. What encourages you to search and read posts?
8. What prevents/discourages you from searching and reading posts?

Prompts if not covered in participant’s responses:

- Physical capability:
  - Are there any physical limitations (e.g., vision, typing ability) that prevent you from using the discussion boards? If yes, can you explain a bit more about that?
- Psychological capability:
  - What skills do you think are important in creating a post / responding to posts (e.g., skills in understanding the original post and replying with relevant information, ability to reply clearly and respectfully)? Do you think you have these skills?
  - Do you think previous knowledge is needed to create a post / respond to posts? Can you give an example or elaborate more?
- Social opportunity
  - Do others (e.g., other users, moderators, friends, family) affect whether you use discussion boards?
- Physical opportunity
  - How do the characteristics or design of the discussion board (e.g., able to access on mobile phone, anonymity, layout) affect your usage of it? How might this be changed/improved to work better for you?
- Automatic motivation
  - How do you feel when creating posts on the discussion boards (e.g., do you have any worries or concerns based on past experience, or feel relieved)? How about for replying posts? Do you feel the same when searching and reading information on the discussion boards?
- Reflective motivation
  - What do you think are the (main) benefits of using discussion boards? Are there any disadvantages you can think of?
  - Do you feel optimistic about whether creating a post / responding to posts / searching and reading information on the discussion boards will help you?
  - What do you expect to see as a result of creating a new post/responding to posts/reading posts? (optional)
  - What would make you more confident in creating a post / responding to posts?

Other questions:

- What do you think are characteristics of a good post and a good reply?
- Are there any other factors that we haven’t covered which influence whether you search and read, post or reply to posts in online forums?
- On the whole, what do you think of The Mix’s discussion boards service? Do you have any suggestions to improve the service?
