# Supplementary material for: Barriers and Enablers to Young People’s Posting, Responding, and Reading Behaviors on Mental Health Forums Using the Behavior Change Wheel: Qualitative Study
Source: JMIR Hum Factors. 2025 Oct 31;12:e71549. doi: 10.2196/71549 (PMC12619017; doi:10.2196/71549)
Supplement: Multimedia Appendix 2 [file humanfactors_v12i1e71549_app2.docx]

This appendix shows the codebook used during the data collection phase. There were two rounds of iterations to the codebook during the coding process. Changes made in these two rounds are indicated with different font colours. Words in red font indicate additions made after the first iteration, while words in green font indicate additions made after the second iteration.

This is an example of a data fragment which was coded differently after discussions amongst the research team: “*The trickier thing, and this is where you might see kind of some threads that take longer to get a reply is when they have like loads and loads of paragraphs. And like, you know,* ***all written in like massive blocks of text. It just it's so much content to go through****.*” Originally, the main researcher coded the bolded phrase under “memory, attention and decision processes”, using the assumption that the length of posts would affect the ability to process the post and reply to it. However, after discussions, the research team agreed that to minimise assumptions made about the availability of cognitive resources, that phrase should be coded under “environmental context and resources” instead. This is because the quote speaks about the physical characteristics of posts and the participant did not explicitly mention any cognitive constraints about processing the long posts.

| **Domain** | **Official definition** | **Definition in this research project's context** | **Examples** | **What it is not** |
| --- | --- | --- | --- | --- |
| Knowledge | An awareness of the existence of something | Being aware of the existence of the function to reply posts in discussion boards, having knowledge related to the activity of responding to posts | - Knowing that there is a function to reply or react to posts in the discussion boards - Knowing how to use the function of replying posts on discussion boards  - Having similar experiences to the person who posted  - Knowing forum guidelines | - Ability to write coherently or succinctly (code under "skills") |
| Skills | An ability or proficiency acquired through practice | Physical or psychological ability/proficiency in replying posts in discussion boards | - Vision or typing ability which may affect ability to reply posts,  - Skills in understanding the original post and replying with relevant information, ability to reply clearly and respectfully | - Emotional feelings (e.g., pride, happiness) about own competency level (code under "emotions") - Awareness about functions of the discussion boards (code under "knowledge") |
| Memory, attention and decision processes | The ability to retain information, focus selectively on aspects of the environment and choose between two or more alternatives | Having the ability to focus on the content of posts on discussion boards and choose whether to reply the posts | - Being able to concentrate on relevant cues and ignore irrelevant cues (e.g., grammatical errors) to reply posts on discussion boards  - Being able to set aside feelings and focus objectively on helping others | - External or system constraints which impact the behaviour of replying to posts on discussion boards (code under "environmental context and resources") |
| Behavioural Regulation | Anything aimed at managing or changing objectively observed or measured actions | Being aware of one's own behaviour of replying and actively planning to reply posts on discussion boards | - Establishing specific processes/systems to help one in replying to posts on discussion boards | - Deciding to reply to posts on discussion boards (code under "intentions") |
| Social/ professional role and identity | A coherent set of behaviours and displayed personal qualities of an individual in a social or work setting | Commitment to reply or not reply in discussion boards due to one's organisational/professional/group identity | - Feeling an obligation to reply because of professional/organisational responsibilities (e.g., being a moderator in the forums), or other social and interpersonal affiliations (e.g., gender, ethnicity) | - Other people's opinion influencing the decision on whether to reply posts on discussion boards (code under "social influences") |
| Beliefs about capabilities | Acceptance of the truth, reality or validity about an ability, talent, or facility that a person can put to constructive use | Beliefs about one's ability to reply posts in discussion boards | - Believing it is easy to reply posts on discussion boards  - Trust in one's own abilities to understand the original posts  - Believing in one’s own abilities to provide constructive/effective reply to posts | - Believing that replying posts will lead to positive outcomes (code under "beliefs about consequences") |
| Optimism | The confidence that things will happen for the best or that desired goals will be attained | Having confidence that outcomes will be for the best with regard to replying posts in the discussion boards | - Believing that things will turn out for the best whether or not one replies to posts in the discussion boards | - Feelings (e.g., anxiety or stress) about replying posts (code under "emotion")- Beliefs about what will happen after replying to posts (code under "beliefs about consequences") |
| Beliefs about consequences | Acceptance of the truth, reality or validity about outcomes of a behaviour in a given situation | Beliefs about certain outcomes that will/will not happen as a result of replying posts on the discussion boards | - Believing that replying posts on discussion boards will help others - Anticipating negative consequences of providing reply that is not useful  - Believing that anonymity will prevent judgement when responding  - Believing that others will judge one’s responses | - Confidence that goals will be achieved (code under "optimism") - Beliefs about whether one has the ability to provide a good reply (code under "beliefs about capabilities") |
| Intentions | A conscious decision to perform a behaviour or a resolve to act in a certain way | Making a conscious decision to reply posts in discussion boards | - Having the resolve or motivation to continue replying to posts (in spite of other influences) | - Establishing practical plans or specific targets/systems/processes to help one in replying to posts on discussion boards (code under "behavioural regulation") |
| Goals | Mental representations of outcomes or end states that an individual wants to achieve | Responding to posts in discussion boards fits in within the end state in which one is striving towards | - Replying to posts on discussion boards aligns with broader goal to help others (e.g., provide emotional support or advice, correct misinformation) | - Establishing practical plans or specific targets/systems/processes to help one in replying to posts on discussion boards (code under "behavioural regulation") - Deciding to reply to posts on discussion boards (code under "intentions") |
| Reinforcement | Increasing the probability of a response by arranging a dependent relationship, or contingency, between the response and a given stimulus | Incentives or disincentives to reply posts in discussion boards | - Rewards, incentives or punishments that come with replying posts on discussion boards (e.g., receiving words of affirmation when responding) | - Beliefs about what would happen instead of things that actually already happened because of replying to posts on discussion boards (code under "beliefs about consequences") |
| Emotion | A complex reaction pattern, involving experiential, behavioural, and physiological elements, by which the individual attempts to deal with a personally significant matter or event | Mood or feelings experienced when replying to posts on discussion boards | - Anxiety or worry about providing wrong advice, or feelings of relief or satisfaction from providing a helpful reply  - Lack of trust in online platforms | - Descriptions of other users' feelings towards receiving a response (code under "social influences") |
| Environmental context and resources | Any circumstance of a person's situation or environment that discourages or encourages the development of skills and abilities, independence, social competence, and adaptive behaviour | External situational or environmental factors (e.g., material resources) that encourages or discourages one from replying to posts on discussion boards | - Ability to access content on different platforms (e.g., mobile phone, website, app)  - Anonymity  - Layout on webpage/forums (e.g., categorisation)  - Length of post | - Influences that are based on interpersonal factors or relationships (code under "social influences") |
| Social influences | Those interpersonal processes that can cause individuals to change their thoughts, feelings or behaviours | Social/group norms or pressure that influence whether one replies to posts on discussion boards | - Views and opinions of others (e.g., friends, family) influencing decision to reply to posts on discussion boards  - Behaviours of other users (e.g., how supportive or negative they are)  - Feelings of similarity to others who are using the forums | - Feeling that one is the type of person who would reply to posts because of affiliation to certain social groups (code under "social/professional role and identity") |
