# Supplementary material for: Barriers and Enablers to Young People’s Posting, Responding, and Reading Behaviors on Mental Health Forums Using the Behavior Change Wheel: Qualitative Study
Source: JMIR Hum Factors. 2025 Oct 31;12:e71549. doi: 10.2196/71549 (PMC12619017; doi:10.2196/71549)
Supplement: Multimedia Appendix 3 [file humanfactors_v12i1e71549_app3.docx]

***Reading***

The credibility of forums and diversity of posts were enablers that encouraged young people to read posts on youth mental health forums. A few participants mentioned that they were more likely to read posts if the forum was backed by a reputable and well-functioning mental health organisation. As explained by P2, a sense of trust and connection could be established as “*there’s an organisation behind this and there’s a team that’s working on this*”. This sense of credibility further stemmed from the perception that “*the information here (is) authentic*” (P12). Many participants also highlighted that the wide variety of posts on youth mental health forums facilitated reading behaviour as it enabled them to gain exposure to “*a range of insights from various groups of individuals and their lives*” (P2), which helped to provide more opportunities for learning: “*one striking benefit would be… learning from each other*” (P10).

***Posting***

For some young people, having the goal of obtaining personalised support to address their unique concerns encouraged them to post in youth mental health forums. They felt that creating a new post would better enable them to receive advice relevant to their problems. For example, one participant highlighted that they would usually create new posts as “*it felt more tailored to me and my current situation*” (P6), while another elaborated that creating a post can result in “*getting many responses and perspectives from different people, giv(ing) me the ideas of what they're thinking and how I can actually use the information they're giving me to solve my issue*” (P11).

***Responding***

Experience in the topic area and having the goal of supporting others were key factors that facilitated responding behaviour. Majority of the young people shared that having personally encountered the situation mentioned in the original post facilitated their responding behaviour as having similar experience enabled them to provide more relevant responses to the original poster: “*If it's a topic that I think is something that I could relate to, I've been through, I can provide some support or some guidance on*” (P2). Having a goal of supporting others on the forum also encouraged some young people to provide emotional support (e.g., to let them know “*they aren't alone*” (P1)) or informational support (e.g., correcting misinformation: “*when I think that someone has posted something that somehow is mythical and has no truth*” (P11)). Furthermore, some were motivated to respond even if they did not have relevant knowledge: “*if someone hasn’t had a reply, I try to reply to them even if it isn’t like helpful but just to let them know that they’ve been listened to and noticed*” (P7). This is because they felt that the purpose of the forums was “*all about helping each other*”
